# The monomeric but not homotrimeric spike protein of SARS-CoV-2 activates TLR4 signaling

**DOI:** 10.1007/s11033-025-10736-4

**Published:** 2025-06-23

**Authors:** Charlotte Lübow, Günther Weindl

**Affiliations:** Pharmaceutical Institute, Section of Pharmacology and Toxicology, University of Bonn, Gerhard-Domagk-Straße 3,, 53121 Bonn, Germany

**Keywords:** SARS-CoV-2 Spike protein, TLR4 signaling, Innate immunity, Pro-inflammatory response, COVID-19 pathogenesis

## Abstract

**Background:**

Recent findings indicate that hyperinflammatory responses to SARS-CoV-2 are major contributors to the severity and fatality of COVID-19. Pattern recognition receptors, particularly Toll-like receptors 2 (TLR2) and 4 (TLR4), have been implicated in detecting SARS-CoV-2 proteins, especially the spike protein. However, the role of viral structural components in triggering innate immune responses remains poorly understood.

**Methods and results:**

HEK293 reporter cells, engineered to stably express TLR2 or TLR4, and THP-1 differentiated macrophages were exposed to various spike protein components and SARS-CoV-2 variants. Protein levels of cytokines were detected by ELISA and mRNA expression was evaluated by quantitative real-time RT-PCR. We demonstrate that the S1 subunit and full-length spike protein elicit TLR4-dependent pro-inflammatory responses. TLR4 activation was triggered by the monomeric, but not the homotrimeric, form of the SARS-CoV-2 spike protein.

**Conclusions:**

These findings suggest that distinct elements of the SARS-CoV-2 spike protein differentially activate TLR4 signaling pathways, driving innate immune and inflammatory responses.

**Supplementary Information:**

The online version contains supplementary material available at 10.1007/s11033-025-10736-4.

## Introduction

Corona virus disease 2019 (COVID-19), caused by the severe acute respiratory syndrome coronavirus 2 (SARS-CoV-2), is a multisystemic disease [1, 2]. The spike protein of SARS-CoV-2 is a key component in the pathogenesis of COVID-19 and serves as a critical target for vaccine development [3]. This protein consists of two different subunits, S1 and S2, which are essential for viral entry into host cells. The S1 subunit binds to the angiotensin-converting enzyme 2 (ACE2) receptor, while the transmembrane protease serine 2 (TMPRSS2) primes the spike protein for cleavage by host proteases. The S2 subunit subsequently facilitates viral fusion with the host membrane, allowing the viral genome to enter the cell and initiate replication.

Severe manifestations of COVID-19 are often linked to an excessive immune response, known as a ‘cytokine storm’, characterized by an overproduction of pro-inflammatory molecules [4, 5] Toll-like receptors (TLRs), which are activated by pathogen-associated molecular patterns (PAMPs), play a crucial role in orchestrating the innate immune response to infection, stress, and tissue damage [6, 7]. TLR2 and TLR4 have been identified as key receptors in recognizing SARS-CoV-2 proteins, thereby triggering pro-inflammatory cytokine release [8–12]. TLR4 activation, in particular, has been implicated in the increased inflammatory response seen in COVID-19 patients [13], as well as in cognitive impairments associated with post-COVID-19 syndrome [14]. However, the precise role of viral structural components in activating TLR-mediated innate immune pathways remains poorly understood. Here, we compared various SARS-CoV-2 spike proteins and uncovered significant differences in their ability to modulate TLR4 signaling.

## Materials and methods

### Cell culture

THP-1 and HEK293 reporter cells were maintained according to previously established protocols [15, 16]. HEK-Blue hTLR2 (hkb-htlr2) and HEK-Blue hTLR4 cells (hkb-htlr4, both from InvivoGen, Toulouse, France) were cultured in growth medium consisting of DMEM (P04-03500, PAN-Biotech GmbH, Aidenbach, Germany) supplemented with 2 mM L-glutamine (G7513), 100 U/ml penicillin, 100 µg/ml streptomycin (P4333, both from Sigma-Aldrich, Taufkirchen, Germany), and 100 µg/ml normocin (ant-nr-1, InvivoGen). Prior to use, HEK-Blue Selection (hb-sel, InvivoGen) was added. HEK-Blue Null1 (hkb-null1) and HEK-Blue Null2 (hkb-null2, both from InvivoGen), which serve as parenteral control cell lines for HEK-Blue hTLR2 and hTLR4, respectively, were used as negative control and cultured in growth medium containing 100 µg/ml zeocin (ant-zn-1, InvivoGen). THP-1 cells (ACC 16, DSMZ-German Collection of Microorganisms and Cell Cultures GmbH, Braunschweig, Germany) were grown in RPMI 1640 with 2 mM L-glutamine (21875091, Thermo Fisher Scientific, Darmstadt, Germany), supplemented with 100 U/ml penicillin, 100 µg/ml streptomycin (P4333, Sigma-Aldrich) and 10% heat-inactivated fetal bovine serum (FBS; S0615, Biochrom, Berlin, Germany). Cells were maintained at a density of 2–8 × 10^5^ cells/ml and cultured at 37 °C in a humidified atmosphere with 5% CO_2_ and 95% air. Cells were used between passages 6 and 25, and were regularly screened for mycoplasma contamination (11-8100, Minerva Biolabs, Berlin, Germany). To differentiate THP-1 monocytes into macrophages, cells were seeded in 24-well plates at a density of 4 × 10^5^ cells/ml and incubated with 25 ng/ml PMA (phorbol 12-myristate 13-acetate; P1585, Sigma-Aldrich) in growth medium. After 48 h, adherent cells were washed with PBS (phosphate buffered saline; 14190144, Thermo Fisher Scientific), and allowed to rest in PMA-free medium for 24 h. The differentiation of these cells was confirmed by expression of differentiation markers and their response to lipopolysaccharide (LPS), as described [16].

### Cell stimulation

HEK-Blue cells were seeded into 96-well plates at a density of 4 × 10^5^ cells/ml in growth medium supplemented with the indicated selective antibiotics. After 24 h, the medium was replaced and cells were stimulated with either the TLR2/6 agonist Pam_2_CSK_4_ (1 ng/ml; tlrl-pm2s-1), the TLR4 agonist LPS (10 ng/ml; tlrl-pb5lp, all from InvivoGen), or various concentrations of recombinant SARS-CoV-2 spike proteins using stimulation medium composed of DMEM and 2 mM L-glutamine. The following recombinant SARS-CoV-2 spike proteins (all from Bio-Techne GmbH, Wiesbaden, Germany) were used: S1 subunit (10569-CV-100), S2 subunit (10594-CV-100), spike protein (10549-CV-100), spike (GCN4-IZ) protein (10561-CV-100), B.1.1.7 spike (GCN4-IZ) protein (10796-CV-100), and B.1.617.2 spike (GCN4-IZ) protein (10878-CV-100). THP-1 macrophages were similarly stimulated with the TLR agonists or SARS-CoV-2 spike proteins (133 nM) in RPMI medium containing 2 mM L-glutamine. In selected experiments, cells were preincubated for 1 h with the TLR2 antagonist MMG-11 (50 µM; 6858/10) or the TLR4 antagonist TAK-242 (2.8 µM; 6587/5, both from Bio-Techne GmbH). To determine the effect of endotoxin contamination on TLR2 or TLR4 activation, the SARS-CoV-2 proteins were subjected to heat inactivation by boiling for 45 min at 100 °C.

### SEAP-reporter assay

A 20 µl sample of cell culture supernatant from HEK-Blue cells was collected and combined with 200 µl of secreted embryonal alkaline phosphatase detection medium (rep-qbs, InvivoGen) in a 96-well plate. The mixture was incubated at 37 °C for 30 min. After incubation, optical density (OD) was measured at 620 nm using a microplate reader (Mitras^2^ LB 943, Berthold Technologies GmbH & Co. KG, Bad Wildbad, Germany).

### ELISA

Following 4 h of stimulation, cell culture supernatants were collected, and cytokine secretion was analyzed using commercially available ELISA kits (CXCL8/IL8 kit, 88-8086-88, Thermo Fisher Scientific).

### RNA isolation, cDNA synthesis, and qRT-PCR

Total RNA extraction, cDNA synthesis, and quantitative real-time RT-PCR (qRT-PCR) were conducted as previously outlined [17, 18]. Primers (synthesized by Eurofins Genomics GmbH, Hamburg, Germany) were used with the following sequences: *GAPDH*, 5’- CTCTCTGCTCCTCCTGTTCGAC-3’ and 5’- TGAGCGATGTGGCTCGGCT-3’; *CXCL8*, 5’- CAAGAGCCAGGAAGAAACCA-3’ and 5’-GTCCACTCTCAATCACTCTCAG-3’; *IFNB*, 5’-CAGCAATTTTCAGTGTCAGAAGC-3’ and 5’-TCATCCTGTCCTTGAGGCAGT-3’; *IL1B*, 5’-TGGAGCAACAAGTGGTGT-3’ and 5’-TTGGGATCTACACTCTCCAGC-3’; *TNF*, 5’-CCCAGGGACCTCTCTCTAATCA-3’ and 5’-GCTACAGGCTTGTCACTCGG-3’. Gene expression was normalized to the housekeeping gene *GAPDH*, which showed the most consistent expression levels. The data was analyzed using the DDCt-method (qPCR soft 4.0, Analytik Jena AG, Jena, Germany). The qRT-PCR reactions, consisting of the cDNA template, primers, and SYBR Green (iTaq Universal SYBR Green Supermix; Bio-Rad, 172–5125) were run under the previously established conditions using the qPCR system qTOWER³G touch (Analytik Jena).

### Statistical analysis

Data are presented as the mean + SEM. Statistical significance for multiple comparisons was assessed using one-way ANOVA followed by a Bonferroni post-hoc test. Significance levels were defined as *P < 0.05, **P < 0.01, ***P < 0.001, ****P < 0.0001. Statistical analysis was performed using GraphPad Prism 8.0 (GraphPad Software Inc., San Diego, CA, USA).

## Results and discussion

**Fig. 1:**
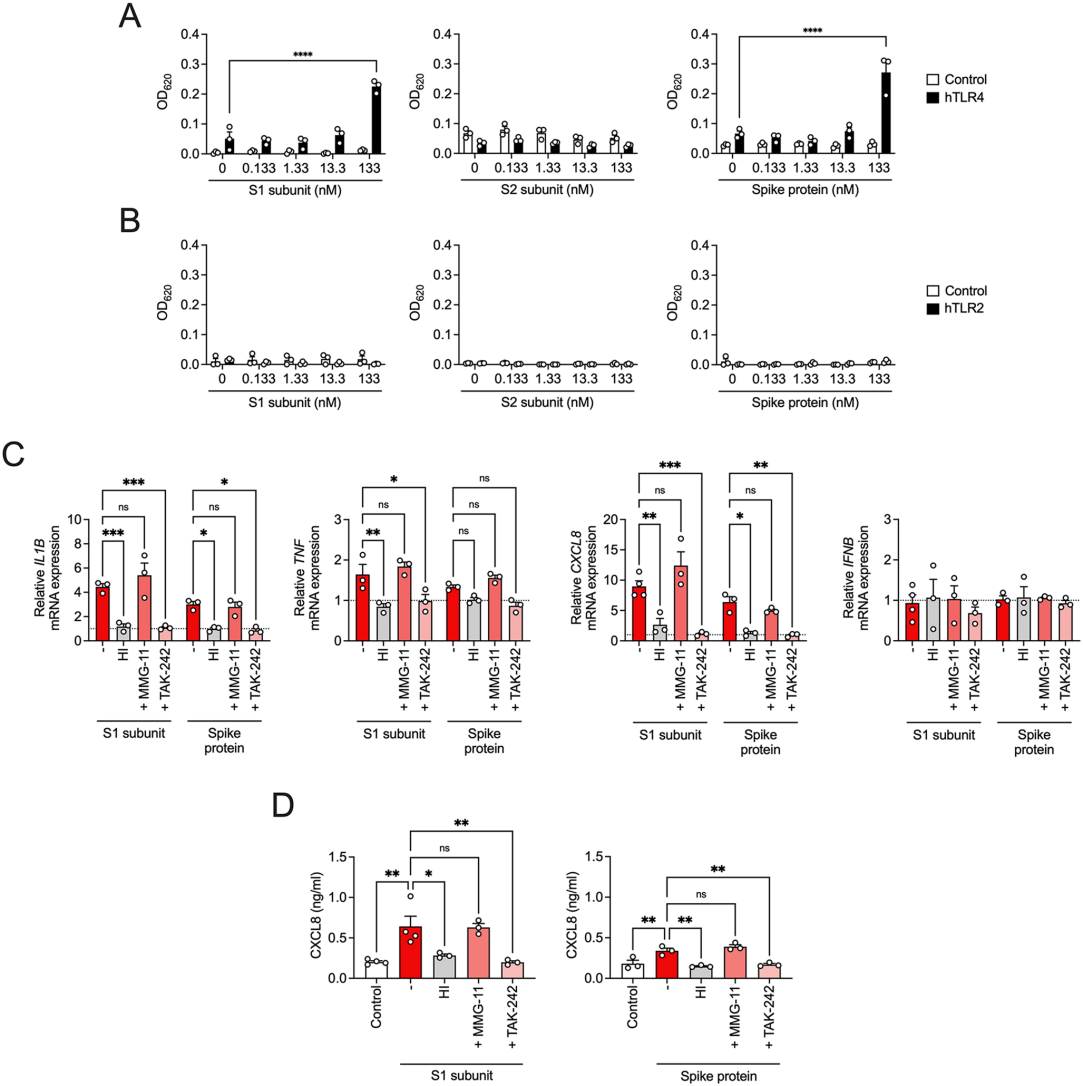
S1 subunit of SARS-CoV-2 and monomeric spike protein trigger TLR4 Signaling. (**A**) hTLR4- and (**B**) hTLR2-HEK293 reporter cells, or the corresponding control cell lines were incubated with different concentrations of recombinant SARS-CoV-2 spike protein S1 subunit, S2 subunit or full-length spike protein for 24 h. SEAP secretion was measured at 620 nm. Mean + SEM (*n* = 3). (**C**, **D**) THP-1 macrophages were preincubated for 1 h with or without the TLR2 antagonist MMG-11 (50 µM) or the TLR4 antagonist TAK-242 (2.8 µM), followed by stimulation with recombinant SARS-CoV-2 spike protein S1 subunit or full-length spike protein (each at a concentration of 133 nM), or the corresponding heat-inactivated proteins (HI) for 4 h. (**C**) Gene expression of *IL1B*, *TNF*, *CXCL8*, and *IFNB* were normalized to the housekeeping gene *GAPDH* (control assigned as 1.0, dotted line). (**D**) Cell culture supernatants were analyzed by ELISA for CXCL8. Mean + SEM (*n* = 3–4). *P < 0.05, **P < 0.01, ***P < 0.001, ****P < 0.0001; ns, not significant; ANOVA followed by Bonferroni posttest

In line with previous reports [9], we observed that the S1 subunit of the SARS-CoV-2 spike protein activated hTLR4-HEK293 reporter cells (Fig. 1A). Similarly, the full-length spike protein induced TLR4-dependent responses, whereas the S2 subunit had no effect. None of the SARS-CoV-2 spike protein forms tested induced signaling through TLR2 (Fig. 1B). These findings were further supported by experiments in THP-1 macrophages, where both the S1 subunit and the full-length spike protein upregulated the gene expression of pro-inflammatory mediators, including interleukin (IL-)1β, tumor necrosis factor (TNF), and C-X-C motif chemokine ligand (CXCL) 8, whereas expression of interferon (IFN) β remained unchanged (Fig. 1C). The pro-inflammatory response was inhibited by the TLR4 antagonist TAK-242, but not by the TLR2 antagonist MMG-11 (Fig. 1C). Consistently, CXCL8 secretion was completely inhibited in the presence of TAK-242 (Fig. 1D). The pro-inflammatory response to the spike protein, as well as the S1 subunit in THP-1 macrophages was not due to endotoxin contamination, as heat-inactivated spike proteins had no effect (Fig. 1C, D). Both TLR antagonists MMG-11 and TAK-242 were confirmed to antagonize TLR2- and TLR4-mediated signaling, respectively (Supplementary Fig. 1A, B). The conformational state of SARS-CoV-2 spike protein is described as homotrimeric, with the S1 subunit being composed of the N-terminal domain (NTD) followed by the receptor binding domain (RBD) and two structurally conserved subdomains protecting the prefusion conformation of the S2 subunit [19]. While neither the single NTD nor the RBD induced pro-inflammatory effects in macrophages [8], our findings indicate that the S1 subunit, consistent with earlier findings [9], and the full-length spike protein activate TLR4-dependent pro-inflammatory responses.

**Fig. 2:**
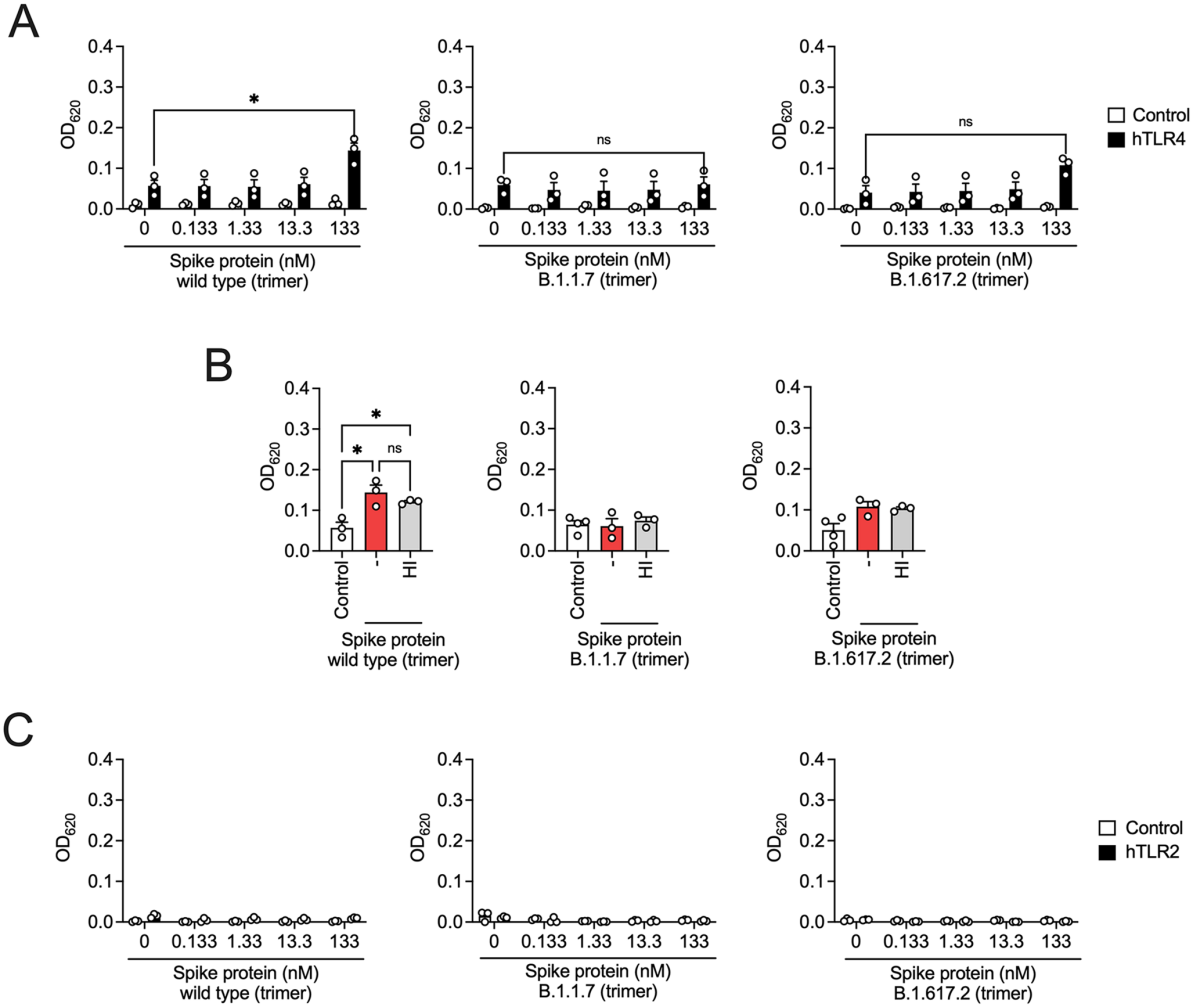
Homotrimeric spike protein of SARS-CoV-2 and variants of concern fail to activate TLR2 and TLR4 signaling. (**A**) hTLR4- and (**C**) hTLR2-HEK293 reporter cells and the corresponding control cell lines were incubated with different concentrations of trimeric recombinant SARS-CoV-2 spike protein wild type, B.1.1.7 (alpha variant), or B.1.617.2 (delta variant) for 24 h. (**B**) hTLR4-HEK293 reporter cells were incubated with trimeric recombinant SARS-CoV-2 spike protein wild type, B.1.1.7 (alpha variant), or B.1.617.2 (delta variant) or the corresponding heat-inactivated proteins (HI) (each at a concentration of 133 nM) for 24 h. SEAP secretion was measured at 620 nm. Mean + SEM (*n* = 3). *P < 0.05; ns, not significant; ANOVA followed by Bonferroni posttest

Previous studies have demonstrated that the trimeric spike protein activates TLR4 [8]. To ensure a predominantly trimeric conformation, we used commercially available recombinant spike proteins engineered with the GCN4-based isoleucine zipper (GCN4-IZ) trimerization domain [20] to stabilize their trimeric structure. Given the global spread of new SARS-CoV-2 variants such as B.1.617.2 (delta), which are associated with higher transmission rates and more severe clinical outcomes, we studied the effects of B.1.17 (alpha) and B.1617.2 (delta) variants compared to the originally variant first identified in Wuhan, China in December 2019 (wild type). The wild-type spike protein showed only a modest increase in TLR4 activation, likely due to residual endotoxin contamination, while the alpha and delta variants showed no increased TLR2- and TLR4-mediated activity (Fig. 2A, B). Also, no induction of pro-inflammatory cytokines was observed in THP-1 macrophages (Supplementary Fig. 2A, B). These findings suggest that TLR4 activation is induced by the monomeric form of the spike protein (Fig. 1), but not by the trimeric form stabilized with the GCN4-IZ domain. The trimeric conformation of the spike protein is essential for binding to the host receptor ACE2; however, dissociation into monomeric S1-ACE2 complexes has been observed [21]. This suggests that monomeric SARS-CoV-2 spike S1 subunits may engage TLR4 in the post-fusion state of the virus.

Comparison of TLR gene expression levels between healthy individuals and COVID-19 patients revealed increased expression of TLR2 and TLR4, which correlated with disease severity [10]. The same study did not show an influence of blocking TLR4 on the pro-inflammatory responses induced by SARS-CoV-2 in peripheral blood mononuclear cells, while a TLR2 antagonist was able to inhibit upregulation of the expression of *TNF*, *IFNG*, *IL1A* or *CXCL10* genes. However, the SARS-CoV-2 envelope protein was responsible for TLR2 activation and spike proteins were indispensable, although it is not clear how the envelope protein, which is embedded in the viral membrane with little extracellular interaction, can activate TLRs [22]. On the contrary, another report indicates that the S2 subunit is mainly responsible for TLR2-mediated immune activation [11]. We could not detect the involvement of TLR2 in pro-inflammatory immune responses by SARS-CoV-2 spike proteins; therefore, further research should clarify if and how SARS-CoV-2 interacts with TLR2.

Although we and others have demonstrated that SARS-CoV-2 spike proteins induce *IL1B* gene expression and thus serve as a priming signal in inflammasome activation, the SARS-CoV-2 nucleocapsid protein (N), which encloses the viral genome, directly mediates the assembly of the NLRP3 inflammasome [23]. Consequently, the nucleocapsid protein functions as the second signal that drives inflammasome activation, leading to the maturation and secretion of IL-1β. Excessive IL-1β production can trigger systemic inflammation through the activation of downstream signaling pathways, leading to increased levels of pro-inflammatory cytokines such as TNF, IL-6, and CXCL8. These findings indicate a coordinated, dual-signal mechanism by which distinct SARS-CoV-2 structural proteins contribute to inflammasome activation and amplify the pro-inflammatory milieu observed in severe COVID-19.

## Conclusion

It is well established that SARS-CoV-2 triggers pro-inflammatory immune responses that can exacerbate disease severity. In this study, we show that the monomeric, but not the trimeric, form of the SARS-CoV-2 spike protein activates TLR4, while neither form activates TLR2. This finding contrasts with previous reports suggesting TLR2-dependent activation by the spike protein [11, 12], as well as TLR4 activation by the trimeric spike protein [8]. Differences in spike protein conformation and glycosylation, depending on the source or method of production, may significantly influence its interaction with TLRs. In fact, we demonstrate that incorporating the GCN4-IZ trimerization domain, which stabilizes the trimeric structure, completely abolishes the ability of the spike protein to activate TLR4. These results highlight the critical role of spike protein structure in modulating innate immune recognition and driving inflammatory responses.

## Electronic supplementary material

Below is the link to the electronic supplementary material.

Supplementary Material 1

## Data Availability

Data is provided within the manuscript or supplementary information files.

## Abbreviations

ACE2: Angiotensin converting enzyme 2
COVID-19: Corona virus disease 2019
CXCL: C-X-C motif chemokine ligand
GCN4-IZ: GCN4-based isoleucine zipper
IFN: Interferon
IL: Interleukin
LPS: Lipopolysaccharide
TNF: Tumor necrosis factor
NLRP3: Nucleotide-binding oligomerization domain-like receptor family pyrin domain-containing protein 3
NTD: N-terminal domain
PAMP: Pathogen-associated molecular pattern
PBS: Phosphate buffered saline
PMA: Phorbol 12-myristate 13-acetate
RBD: Receptor binding domain
SARS-CoV-2: Severe acute respiratory syndrome coronavirus 2
TLR: Toll-like receptor
TMPRSS2: Transmembrane protease serine subtype 2
