# Supplementary material for: The monomeric but not homotrimeric spike protein of SARS-CoV-2 activates TLR4 signaling
Source: Mol Biol Rep. 2025 Jun 23;52(1):629. doi: 10.1007/s11033-025-10736-4 (PMC12185570; doi:10.1007/s11033-025-10736-4)
Supplement: Supplementary file 1 — Supplementary Material 1 [file 11033_2025_10736_MOESM1_ESM.pdf]

Supplementary Information

The monomeric but not homotrimeric spike protein of SARS-CoV-2 activates TLR4 signaling

Charlotte Lübow, Günther Weindl*

University of Bonn, Pharmaceutical Institute, Section of Pharmacology and Toxicology, Germany

*e-mail: guenther.weindl@uni-bonn.de.

This PDF file includes:

Supplementary Figures 1 and 2

Supplementary figures

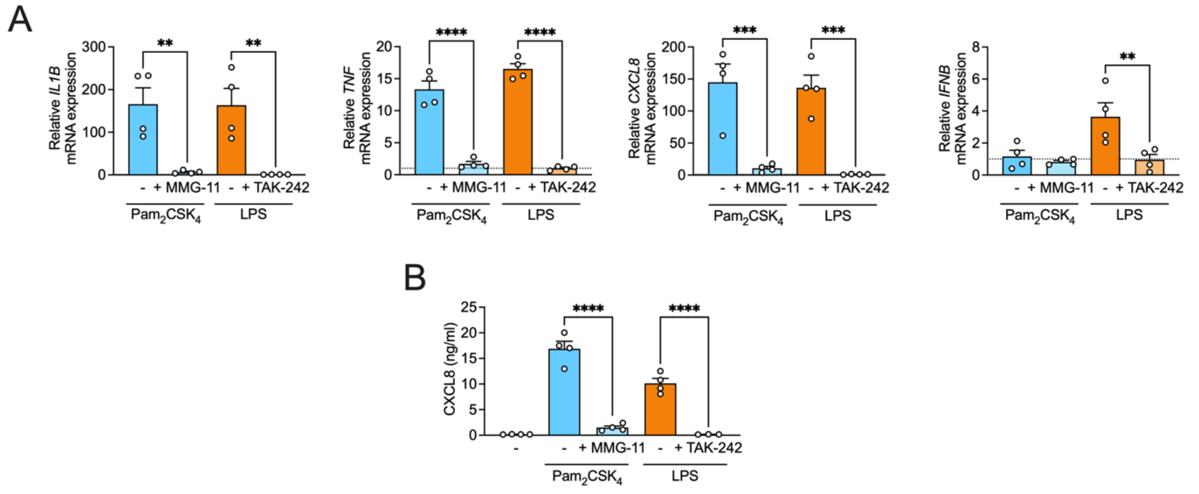

Supplementary Fig. 1. (**A**, **B**) THP-1 macrophages were preincubated with or without the TLR2 antagonist MMG-11 (50 µM) or the TLR4 antagonist TAK-242 (2.8 µM) for 1 h and subsequently stimulated with Pam_2CSK_4 (1 ng/ml) or LPS (10 ng/ml) for 4 h. (**A**) Gene expression of *IL1B*, *TNF*, *CXCL8*, and *IFNB* were normalized to the housekeeping gene *GAPDH* (control assigned as 1.0, dotted line). (**B**) Cell culture supernatants were analyzed by ELISA for CXCL8. Mean + SEM ($n = 4$). **P<0.01, ***P<0.001, ****P<0.0001; ANOVA followed by Bonferroni posttest.

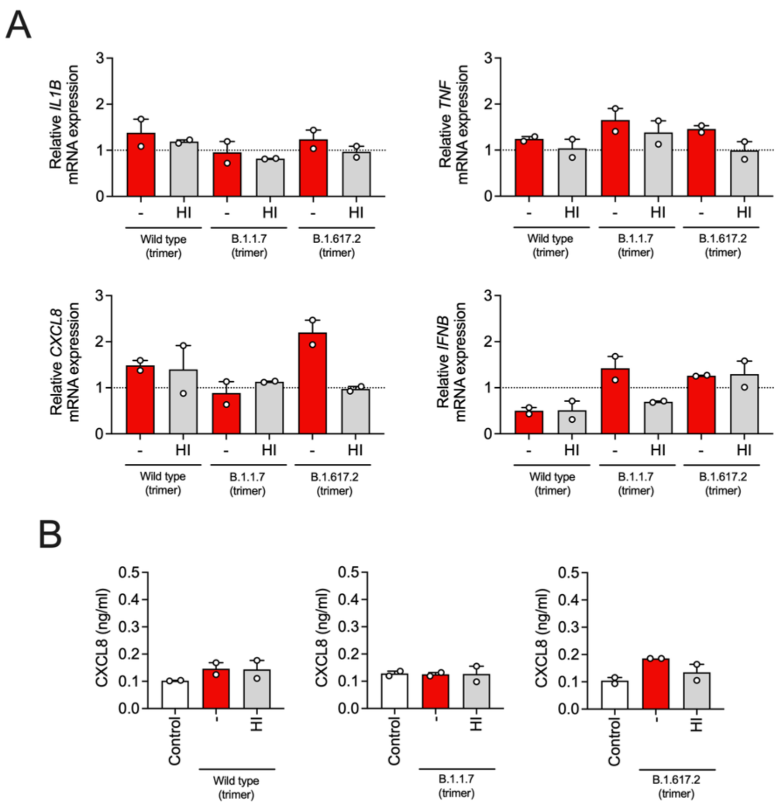

Supplementary Fig. 2. (**A**, **B**) THP-1 macrophages were stimulated with trimeric recombinant SARS-CoV-2 spike protein wild type, B.1.1.7 (alpha variant), or B.1.617.2 (delta variant), or the corresponding heat-inactivated proteins (HI) (each at a concentration of 133 nM) for 4 h. (**A**) Gene expression values of *IL1B*, *TNF*, *CXCL8*, and *IFNB* were normalized to the housekeeping gene *GAPDH* (control assigned as 1.0, dotted line). (**B**) Cell culture supernatants were analyzed by ELISA for CXCL8. Mean + SEM ($n = 2$).
